# Prevention of Liver Fibrosis by Intrasplenic Injection of High-Density Cultured Bone Marrow Cells in a Rat Chronic Liver Injury Model

**DOI:** 10.1371/journal.pone.0103603

**Published:** 2014-09-25

**Authors:** Jie Lian, Yang Lu, Peng Xu, Ai Ai, Guangdong Zhou, Wei Liu, Yilin Cao, Wen Jie Zhang

**Affiliations:** Department of Plastic and Reconstructive Surgery, Shanghai 9^th^ People's Hospital, Shanghai Jiao Tong University School of Medicine, Shanghai Key Laboratory of Tissue Engineering, National Tissue Engineering Center of China, Shanghai, China; Cardiological Center Monzino, Italy

## Abstract

Endothelial progenitor cells (EPCs) from bone marrow have proven to be functional for the prevention of liver fibrosis in chronic liver injury. However, expansion of EPCs in culture is complicated and expansive. Previously, we have established a simple method that could enrich and expand EPCs by simple seeding bone marrow cells in high density dots. The purpose of this study is to evaluate whether cells derived from high-density (HD) culture of rat bone marrow cells could prevent the liver fibrosis in a chronic liver injury rat model, induced by carbon tetrachloride (CCl_4_). Flow cytometric analysis showed that cells from HD culture were enriched for EPCs, expressing high levels of EPC markers. Intrasplenic injection of HD cultured bone marrow cells in the CCl_4_-induced liver injury rat showed an enhanced antifibrogenic effect compared with animals treated with cells from regular-density culture. The antifibrogenic effect was demonstrated by biochemical and histological analysis 4 weeks post-transplantation. Furthermore, cells from HD culture likely worked through increasing neovascularization, stimulating liver cell proliferation, and suppressing pro-fibrogenic factor expression. HD culture, which is a simple and cost-effective procedure, could potentially be used to expand bone marrow cells for the treatment of liver fibrosis.

## Introduction

The liver possesses great regenerative capacity in response to injury. However, chronic injuries caused by autoimmune hepatitis, alcohol abuse, metabolic disorders, or viral hepatitis, could disturb the regenerative process, leading to development of a common pathology known as liver fibrosis [1]. In some cases, persistent injuries progress the fibrosis and eventually lead to liver cirrhosis [1]. At this stage, the only therapeutic option is organ transplantation. Liver transplants are not widely performed because of problems such as donor shortage, surgical invasiveness, risk of immunological rejection, and medical costs [2]. Therefore, it is essential that therapeutic alternatives to liver transplantation are developed.

Recently, the emergence of stem cell research has opened new possibilities for the treatment of chronic liver diseases. Various cell populations from the bone marrow, including hematopoietic stem cells (HSCs) [3]–[5], mesenchymal stem cells (MSCs) [6], [7], endothelial progenitor cells (EPCs) [8], [9], and bone marrow mononuclear cells (BMNCs) [10], have been transplanted and have proved to be functional for the prevention of liver fibrosis in animal models, as well as in patients. The transplanted cells likely play multiple roles in the repair process. They may differentiate directly into hepatocytes, or release growth factors to protect intrinsic hepatocytes, stimulate regeneration, regulate inflammatory response, and/or decompose the extracellular matrix (ECM) [9], [11]–[13]. Although non-cultured autologous bone marrow-derived cells have been successfully applied in patients [7], [10], the use of *in vitro* culture-expanded cells for treatment could reduce the initial amount of bone marrow needed.

Expansion of stem cells in culture is still a big challenge in the field. For example, it is difficult and expensive to expand EPCs without losing their stemness and function [14]. Previously, we established a novel and simple bone marrow high-density (HD) culture system by seeding BMNCs in HD dots on tissue culture plates [15]. Pre-coating of the plates and addition of growth factors are not required using this culture technique. Cells expanded in HD culture display EPC characteristics and have high pro-angiogenic potential. In addition, the cells secrete higher levels of vascular endothelial growth factor (VEGF) and hepatocyte growth factor (HGF), compared with cells grown in regular-density (RD) culture [15]. On the basis of these advantages, we speculate that these cells might be better than cells from RD culture (which contains mainly MSCs), for the treatment of liver fibrosis. To test this hypothesis, the antifibrogenic and regenerative effects of high- and RD cultured bone marrow cells were investigated in a carbon tetrachloride (CCl_4_)-induced rat chronic liver fibrosis model.

## Materials and Methods

### 1. Animals and experimental models

Male Wistar rats (6 weeks old) weighing approximately120 g were purchased from the Shanghai Chuansha Experimental Animal Raising Farm (Shanghai, China). Animal study protocols were approved by The Animal Care and Experiment Committee of Shanghai Jiao Tong University School of Medicine. The liver injury model was created by injections of CCl_4_ (Sigma, St. Louis, MO, USA) as described previously [16]. Briefly, a 10% solution of CCl_4_was prepared in olive oil and a dose of 2 mL/kg was injected intra-peritoneally every other day over 6 weeks. After the 6-week injection course, all rats were submitted to a blood test for serum level of albumin (ALB), glutamic oxalacetic transaminase (AST), and glutamic pyruvic transaminase (ALT), to evaluate liver injury and function. Only those animals presenting blood values different from the predefined range, for all three parameters, were used in the transplantation studies.

### 2. Isolation and culture of bone marrow cells

Rat bone marrow cells were extracted from the femurs of 6-week-old male Wistar rats. To remove the majority of non-adherent blood cells, primary culture of bone marrow cells was performed by seeding the cells at 1.6×10^4^ cells/cm^2^ in Dulbecco's modified Eagle's medium (DMEM; Invitrogen, Carlsbad, CA, USA) with 10% fetal bovine serum (FBS; HyClone, Logan, UT, USA) and 0.2% penicillin/streptomycin(Sigma). Medium was changed every 3 days. After 6–7 days of culture, primary adherent cells (P0) were harvested using trypsin/EDTA (0.25% w/v trypsin, and 0.02% EDTA; Invitrogen), and were subcultured at high or regular density. For RD culture, 9×10^5^ primary cultured cells were seeded evenly at a density of 1.6×10^4^ cells/cm^2^ in a 10-cm diameter tissue culture dish in 10 mL DMEM with 10% FBS. Cells were passaged in the same manner every 3 days. For HD culture, 9×10^5^primary cells (equal to the cell number of RD culture) were suspended in 300 µL of culture medium, and then six drops (50 µL each) of cell suspension were dot-seeded separately onto a 10-cm-diameter culture dish within equal distance. The average diameter of each dot was 1 cm, resulting in a final local cell density of 2×10^5^ cells/cm^2^. Culture dishes were placed in an incubator for 30 min, and then 10 mL of culture medium was gently added to cover each dish. Medium was changed every 3 days. Cells were passaged in the same manner at day 7 and collected for analysis and transplantation at day 15.

### 3. Flow cytometric analyses

After 15 days culture, cells were trypsinized and aliquots of 2×10^5^ cells were suspended in 200 µL washing buffer (PBS containing 2% FBS). Cells were then incubated on ice for 30 min with phycoerythrin (PE)- or fluorescein isothiocyanate (FITC)-conjugated antibodies. PE- and FITC-conjugated isotype matched immunoglobulins were used as controls. After staining and washing, cells were analyzed on a flow cytometer (Epics Altra; Beckman Coulter, Fullerton, CA, USA). Antibodies against the following markers were used: CD29, CD90, CD31 (BD Biosciences, San Diego, CA, USA), CD133, and KDR (Abcam, Cambridge, UK). Flow cytometric data were analyzed with CXP software (Beckman Coulter).

### 4. *In vitro* angiogenesis assay

One hundred μL of Matrigel (BD Biosciences) basement membrane matrix was added to 24-well plates. The plates were then incubated for 30 min to allow gel solidification. Then, 2×10^4^ cells from 15-day HD or RD cultures were seeded onto the gel in 500 µL EGM-2 (Invitrogen). Twelve hours later, the plates were observed under a light microscope (Olympus, Tokyo, Japan). Nine representative fields were recorded and the average number of branch points was calculated by Image-Pro Plus software (Media Cybernetics, Atlanta, GA, USA).

### 5. Cell labeling and transplantation

After 15 days of HD or RD culture, cells were labeled with 1,1′-dioctadecyl-3,3,3′,3′-tetramethylindocarbocyanine dye (CM-DiL; Invitrogen) following the manufacturer's instructions, before transplantation. Rats with liver injury, induced by the 6-week injection course of CCl_4_, were divided into three groups (n = 6/group): (1) HD group; (2) RD group; (3) PBS group. Two million cells from HD or RD cultures were suspended in 200 µL PBS and injected intrasplenic using a 29-G needle. Rats that received the same volume of PBS served as controls. After cell transplantation, CCl_4_ injections were continuously administrated every other day for another 4 weeks. Rats were then sacrificed and all blood samples and livers were harvested. Serum ALB, AST, and ALT were measured according to standard clinical methods.

### 6. Histopathology

Liver tissues were fixed in 4% paraformaldehyde for 12 h, embedded in paraffin and cut into 5-μm sections. Sections were stained with hematoxylin and eosin (HE), picric acid-sirius red (PSR), and Masson, for histological structure analysis and fibrosis area analysis. Five randomly selected fields of view, from PSR- and Masson-stained sections of each sample (n = 3/group), were captured by a light microscope (Olympus, Tokyo, Japan).The fibrosis area was measured using Image-Pro Plus software (Media Cybernetics). The percentage fibrosis area was calculated by comparing the collagen stained area to the total area of the fields examined.

Immunohistochemical (IHC) staining was carried out as described previously using commercially available antibodies against collagen I, α-smooth muscle actin (α-SMA), Ki-67 (all from Abcam), and CD31 (Santa Cruz Biotechnology, Santa Cruz, CA, USA). This was followed by horseradish peroxidase-conjugated goat anti-mouse antibody (Dako, Denmark) and colorized with diaminobenzidine tetrahydrochloride (DAB, Dako) [8]. For blood vessel density analysis, five randomly selected fields, from anti-CD31 staining of each sample (n = 3/group), were captured by a light microscope (Olympus).The number of blood vessels was calculated by Image-Pro Plus software (Media Cybernetics).

### 7. Immunofluorescent analysis of cell distribution

To detect cell distribution in the liver after intrasplenic injection, liver tissues were harvested at 4 weeks post-transplantation, embedded in OTC compound and frozen slides were sectioned at 10 µm. Three mice from each group and three sections from each mouse, were stained with FITC-conjugated anti-collagen type I (Abcam) and DAPI (Invitrogen), and were observed under a confocal microscope (Leica, Solms, Germany). The number of CM-DiL-labeled cells was calculated from five fields of view for each sample by Image-Pro Plus software (Media Cybernetics).

### 8. Quantitative reverse transcription polymerase chain reaction (qRT-PCR)

Total RNA extracted from liver tissues were reverse transcribed into cDNA and subsequently amplified using a Power SYBR Green PCR master mix (2×) (Applied Biosystems) in a real-time thermal cycler (Mx3000PTM QPCR System; Stratagene). Primers are listed in Table 1. qRT-PCR was conducted in triplicate for each sample. Gene expression was normalized to glyceraldehyde-3-phosphate dehydrogenase (GAPDH) expression. Results represent three independent experiments.

**Table 1:** Primers used in qRT-PCR analyses.

Target Gene	Primer (5′-3′)	Sequence (5′-3′)
α_2_-procollagen	Forward	ATGTTCAGCTTTGTGGACCT
	Reverse	CAGCTGACTTCAGGGATGT
Fibronectin	Forward	AGACTGCAGTGACCACCATCC
	Reverse	CAATGTGTCCTTGAGAGCATAGAC
α-SMA	Forward	CGAAGCGCAGAGCAAGAGA
	Reverse	CATGTCGTCCCAGTTGGTGAT
TGF-β	Forward	GAAGGACCTGGGTTGGAAGT
	Reverse	CGGGTTGTGTTGGTTGTAGAG
HGF	Forward	CCTATTTCCCGTTGTGAAG
	Reverse	ACTAACCATCCACCCTACTG
VEGF-A	Forward	CCACACCACCATCGTCAC
	Reverse	CCAGAAACAAAACTCCCTAATC
VEGFR-2	Forward	GCAAATACAACCCTTCAGATTA
	Reverse	CACCCTTTCCTCAGAGTCAC
Ang-1	Forward	GCTGGCAGTACAATGACAGT
	Reverse	TCTGGAAGAATGAAAGTGTAGG
Tie-2	Forward	GATGAAGGGCAAGATGGATAG
	Reverse	AGAAGCAGGCGGTAACAGT
MMP-2	Forward	CAAGTGGGACAAGAATCAGA
	Reverse	GAGAAAAGCGTAGTGGAGTTAC
TIMP-2	Forward	CTTAGCATCACCCAGAAGAAGA
	Reverse	GTCCATCCAGAGGCACTCAT
PDGF-B	Forward	GAGGAGGAGACGGGCA
	Reverse	CACTGAACAAACGGACACT
PDGFR	Forward	TTGTCACGGATGTCACTGAGA
	Reverse	AAACCTCGCTGGTGGTCATA

### 9. Statistical analysis

Data were expressed as the mean ± standard deviation. Comparisons between groups were analyzed by ANOVA. A value of p<0.05 was considered statistically significant.

## Results

### 1. Identification of bone marrow cells after *in vitro* expansion

**Figure 1:**
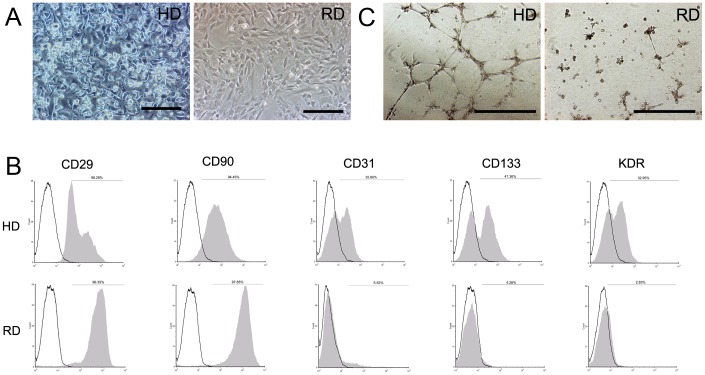
Endothelial progenitor cells enriched in HD cultured bone marrow cells. (A) Morphology of rat bone marrow cells in HD and RD culture. (Scale bars:100 µm). (B) Flow cytometry analyses of HD and RD cultured cells after 15 days of expansion. (C) Tube formation ability of RD and RD cultured cells on matrigel. (Scale bars:200 µm).

The characteristics of rat bone marrow cells after 15 days culture were identical to those previously described [15]. In HD culture, a population of small bright cells growing on top of spindle-shaped cells was observed, while cells in RD culture displayed a spindle-shaped fibroblastic morphology only (Fig. 1A). Flow cytometric analysis showed that cells from HD culture expressed higher levels of CD34, CD133, and FLK-1(KDR) EPC markers, compared with cells from RD culture (Fig. 1B). MSC markers, CD90 and CD29, were highly expressed in both cultures. The *in vitro* tubular formation assay confirmed that cells from HD culture formed obvious tubular networks, which were absent in cells from RD culture (Fig. 1C), indicating that cells from the HD culture contained more EPCs.

### 2. Protective effect of cell transplantation in liver function

**Figure 2:**
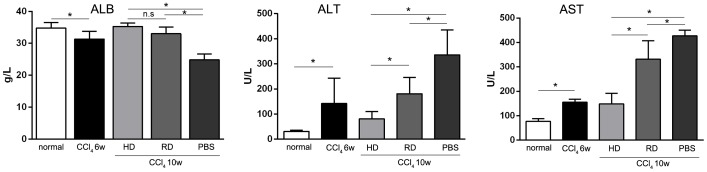
Protective effect of cell transplantation in liver function. Blood samples of normal and experimental rats were collected and serum levels of albumin (ALB), glutamic oxalacetic transaminase (AST) and glutamic pyruvic transaminase (ALT) were tested before (CCl_4_ 6w) and after (CCl_4_ 10w) cell transplantation. (n = 6/group; *p<0.05).

Rats that received 6 weeks of CCl_4_ injection displayed chronic liver injury, with an increased serum level of ALT (58.6±9.56 U/L) and AST (155±10.54 U/L), and a decreased serum level of ALB (32.6±1.48 U/L) (Fig. 2). Four weeks after cell transplantation, blood samples were tested again. As shown in Fig. 2, both ALT and AST were significantly increased in the PBS-treated group, but not in the group that received HD cultured cells. ALT and AST levels in the group that received RD cultured cells were also increased but lower than those in the PBS-treated group. A decreased ALB level was observed in the PBS group but not in the other two cell-transplanted groups. These results indicate that cell transplantation could prevent liver damage to some extent, and HD cultured bone marrow cells performed better than RD cultured cells.

### 3. Antifibrogenic effect of cell transplantation in liver fibrosis

**Figure 3:**
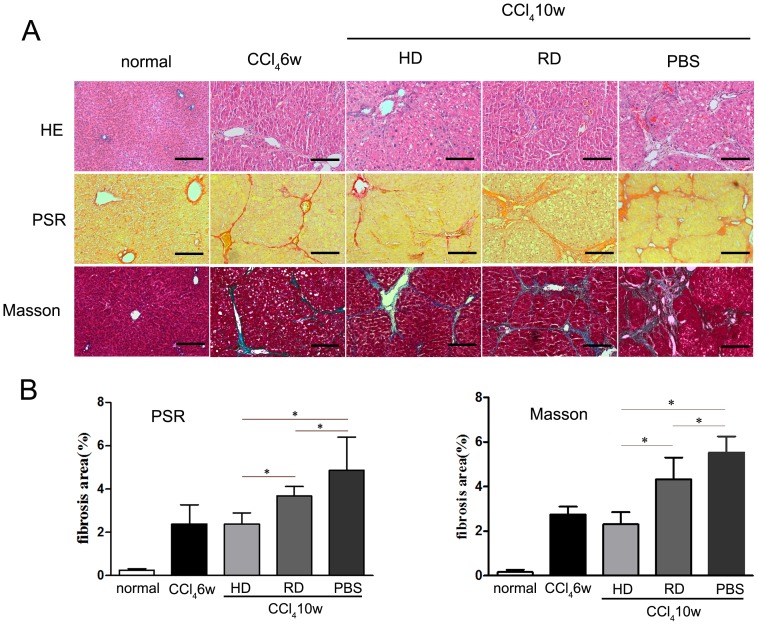
Antifibrogenic effect of cell transplantation in liver fibrosis. (A) Representative structural changes in livers were detected by hematoxylin & eosin (HE), picric acid-sirius red (PSR) and Masson staining (Scale bars: 100 µm). (B) Quantitative analyses of liver fibrosis were performed from PSR and Masson staining. Five views from each sample with three samples in each group were analyzed. *p<0.05.

To evaluate the antifibrogenic effect following cell transplantation, histologic examinations were performed to detect liver structures and ECM deposition. HE staining showed that after 6 weeks of CCl_4_ injection, rat liver tissues formed pseudolobules that remolded the liver morphology (Fig. 3A). After 4 more weeks of CCl_4_ injection, liver structure in the PBS-treated group was further destroyed with wide formation of pseudolobules. However, less pseudolobule structures were observed in both cell-transplanted groups. Morphological changes were confirmed by PSR and Masson staining (Fig. 3A). Quantitative analyses of liver fibrosis were performed from PSR and Masson staining. The fibrotic area increased after 6 weeks of CCl_4_ injection, and continuously progressed in the PBS group. However, fewer fibrotic areas were observed in the cell-transplanted groups with an enhanced antifibrogenic effect observed in the group treated with HD cultured cells (Fig. 3B).

**Figure 4:**
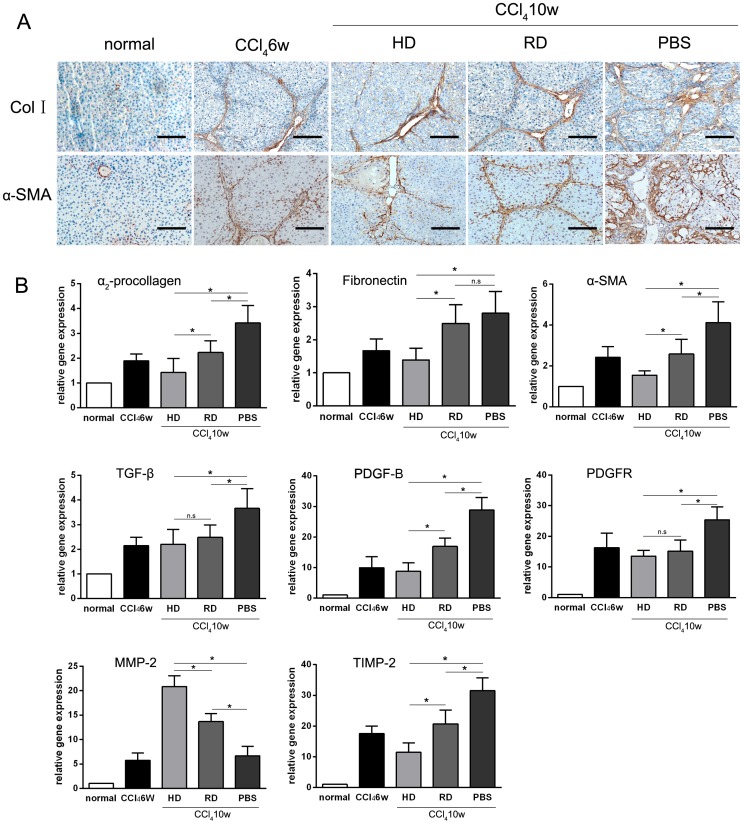
Antifibrogenic effect of cell transplantation measured by immunohistology and qRT-PCR analyses. (A) Immunohistochemical staining of collagen type I (Col I) and α-smooth muscle actin (α-SMA) before (CCl_4_ 6w) and after (CCl_4_ 10w) cell transplantation (Scale bars: 100 µm). (B) Quantitative RT-PCR analysis of fibrosis related markers α_2_-procollagen, fibronectin, α-SMA, TGF-β,PDGF-B, PDGFR, MMP-2 and TIMP-2. Each sample was repeated three times with three samples from each group. *p<0.05; n.s: p>0.05.

To further confirm the antifibrogenic effect of cell transplantation, IHC staining of collagen I and α-SMA were performed. Like PSR and Masson staining, similar changes in liver structure and ECM deposition patterns were observed (Fig. 4A). Quantitative RT-PCR analyses of α_2_-procollagen and α-SMA in liver tissues showed that gene expression levels were lower in both HD and RD groups than those in the PBS group, while the gene expression of fibronectin in HD group was lower than those in RD and PBS groups (Fig 4B). Accompany this, expression of TGF-β, a key pro-fibrogenic growth factor, was decreased in the cell-transplanted groups compared with the PBS group. However, no significant difference was observed between the HD group and the RD group (Fig. 4B). The expressions of another pro-fibrogenic growth factor, platelet-derived growth factor subunit B (PDGF-B), and its receptor (PDGFR), were also decreased in the cell-transplanted groups (Fig. 4B). Matrix metalloproteinase (MMP)-2, which relates to matrix degradation, were up-regulated in the cell-transplanted groups. While, the tissue inhibitor of metalloproteinase (TIMP)-2 were down-regulated in those groups (Fig. 4B).

### 4. Distribution of transplanted cells in liver

**Figure 5:**
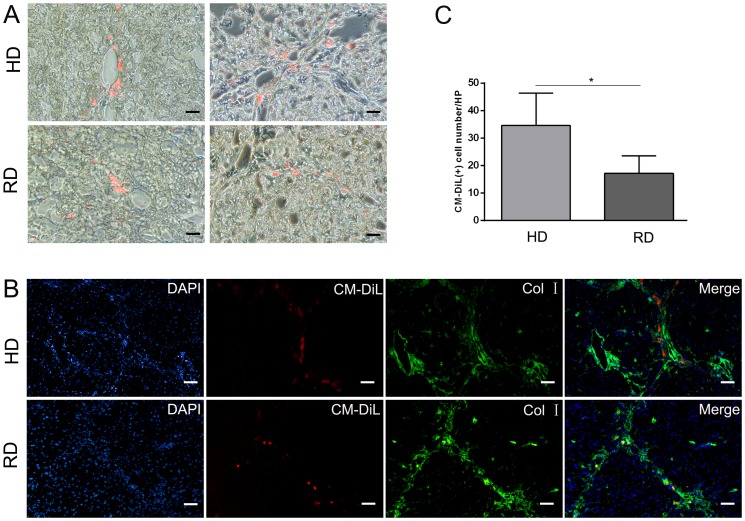
Distribution of transplanted cells in fibrotic liver. (A) Transplanted cells with red fluorescence of 1,1′-dioctadecyl-3,3,3′,3′-tetra-methylindocarbocyanine dye (CM-DiL) were detected under confocal microscope (Scale bars: 50 µm). Positive cell were counted form five views from each sample with three samples in each group. *p<0.05. (B) Immunofluorescence staining of collagen type I (Col I) revealed that CM-DiL positive cells were located around portal vein and fibrous septa (Scale bars: 50 µm).

An enhanced antifibrogenic effect was observed in the group treated with HD cultured cells. To explain this phenomenon, we first detected distribution of the injected cells in the liver 4 weeks after cell transplantation. As shown in Fig. 5, CM-DiL-labeled cells were observed in the liver, with a greater number of cells observed in the HD cultured group (Fig. 5A,C), indicating that more HD cultured cells homed to the liver and survived. Interestingly, the majority of transplanted cells were observed around the portal tracts, fibrous septa, and hepatic sinusoids in both the HD and RD groups (Fig. 5B). In accordance with the IHC staining, less collagen I staining was observed in the HD group than in the RD group (Fig. 5B).

### 5. Promotion of liver regeneration by transplanted cells

**Figure 6:**
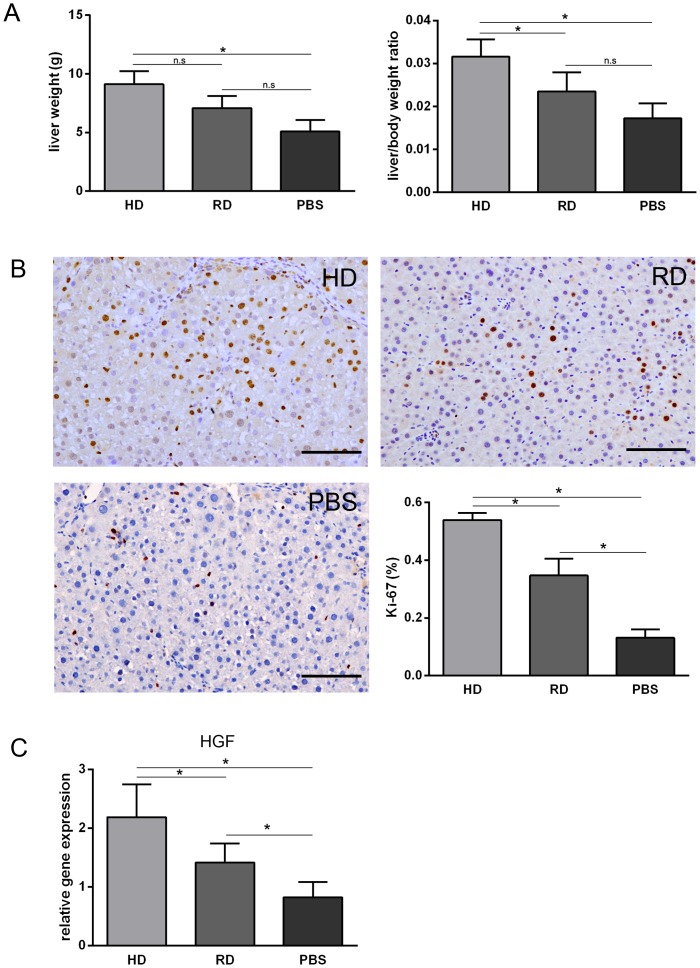
Promotion of liver regeneration by transplanted cells. (A) The liver weights and liver/body weight ratios at 4 weeks post-treatment. (B) Representative views of immunohistological staining of Ki-67 in HD group, RD group and PBS group (Scale bars: 150 µm). Percentage of Ki-67 positive cells calculated from immunohistological staining. Five views from each sample with three samples in each group were analyzed. (C) Expression of hepatocyte growth factor (HGF) analyzed by qRT-PCR. Each sample was repeated three times with three samples from each group. *p<0.05.

It has been widely observed that transplanted cells stimulate liver regeneration through promoting the proliferation of resident hepatocytes. Liver weights and liver/body weight ratios measured at 4 weeks post-treatment showed a higher liver weights and liver/body weight ratios in the cell-transplanted groups compare to those in the PBS-treated group (Fig. 6A). We further performed IHC staining of Ki-67 in the liver sections. More Ki-67 positive cells were observed in the HD group compared with the RD and PBS groups (Fig. 6B). Because cells in the HD culture expressed higher levels of HGF [15], expression of HGF in the liver tissue was examined 4 weeks after cell transplantation. As expected, a higher level of HGF expression was observed in the HD group compared with the other groups (Fig. 6C).

### 6. Increased sinusoidal blood vessel density by transplanted cells

**Figure 7:**
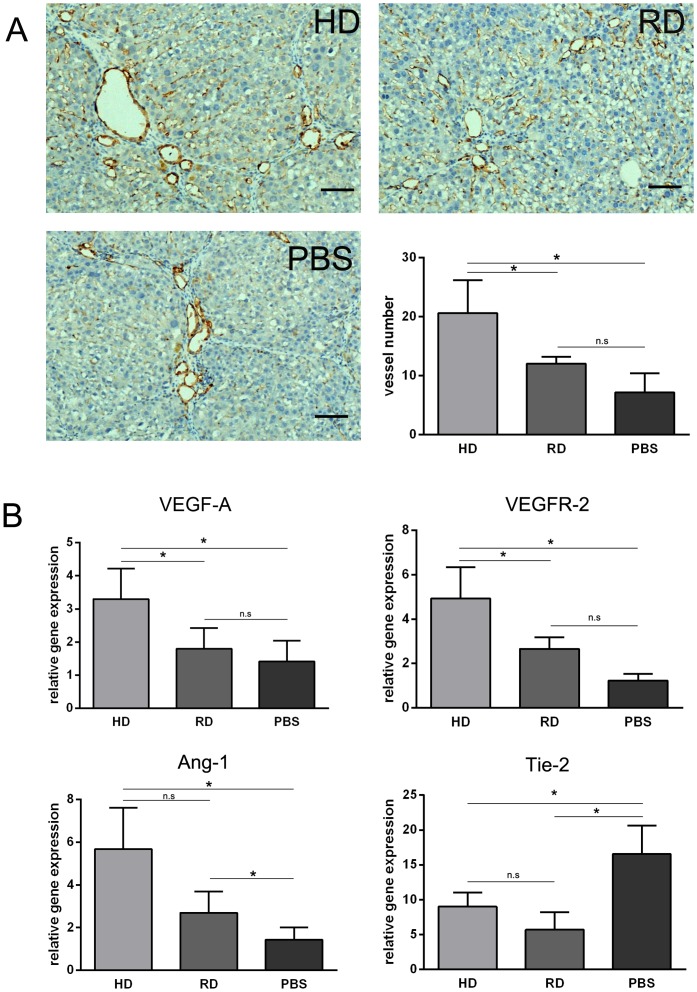
Increased sinusoidal blood vessel density by transplanted cells. (A) Representative views of immunohistological staining of CD31 in HD group, RD group and PBS group (Scale bars: 150 µm). Number of CD31 positive vessels counted from immunohistological staining. Five views in each sample with three samples in each group were analyzed. (B) Expression of Ang-1, Tie-2, VEGF-A and VEGFR-2 analyzed by qRT-PCR. Each sample was repeated three times with three samples from each group. *p<0.05.

In many organs, neovascularization has been demonstrated to be crucial to the healing of injured tissues, which involves mature endothelial cells and EPCs. In many ways, the liver's response to injury involves neovascularization, including new vessel formation and sinusoid remodeling [17]. To measure blood vessel density after cell transplantation, anti-CD31 IHC staining of liver sections was performed. As shown in Fig. 7A, more CD31 positive blood vessels were observed in the HD group than those in the RD and PBS groups. Further qRT-PCR analysis of pro-angiogenic gene expression was consistent with the above observation, that a higher level of VEGF-A and angiopoietin-1 (Ang-1) expression were observed in livers transplanted with HD cultured cells, accompanied with an increased expression of VEGF receptor-2 (VEGFR-2) but decreased expression of Tie-2 (Fig. 7B).

## Discussion

Recent advances in stem cell research have revealed that bone marrow-derived cells, including HSCs, MSCs, EPCs, and BMNCs, could significantly protect liver function after injury [11], [18]. In the present study, we demonstrated that an EPC enriched population from a novel HD culture of bone marrow cells, displayed better antifibrogenic potential in the treatment of chronic liver injury. The antifibrogenic effect was determined by biochemical and histological evidence.

Previously, we demonstrated that EPCs could be expanded in HD culture without pre-coating culture dishes and addition of extra growth factors, which is a simple and cost-effective method for *in vitro* expansion of bone marrow EPCs [15]. On the contrary, the RD culture, which has been adopted widely for MSCs culture [19], could efficiently expand MSCs with loss of EPCs during cell passage (Fig. 1B,C). Although no previous reports have compared the efficacy of EPCs to MSCs in the treatment of chronic liver injury, the current work provides evidence that bone marrow cells enriched for EPCs could function better than relatively pure MSCs *in vivo*. The mechanism of action can be explained as follows: first, more cells from the HD culture homed to and survived in the injured liver after injection (Fig. 5); second, a lower level of the pro-fibrogenic factors, TGF-β and PDGF-B, were expressed in the liver of the HD group (Fig. 4B); third, matrix degradation enzyme MMP-2 were highly expression in HD group (Fig. 4B); fourth, cell proliferation was stimulated through higher expression of HGF in the HD group (Fig. 6); and finally, more blood vessels were formed, induced by higher expressions of VEGF-A and Ang-1 in the HD group (Fig. 7B). Obviously, the secretion of growth factors played a crucial role in the antifibrogenic process, which is consistent with other reports. Interestingly, differentiation of transplanted cells into hepatocytes was not observed (data not shown), indicating that the injected cells functioned mainly through a paracrine mechanism to prevent liver fibrosis and regeneration, rather than by direct differentiation into hepatocytes.

The enhanced antifibrogenic effect of HD cultured bone marrow cells could also be explained by the natural liver repairing mechanism after injury. It is known that in many tissues, the response to injury involves angiogenesis, which requires a supply of growth factors, nutrients, and oxygen. In liver regeneration, resident sinusoidal endothelial cells, have been shown to proliferate, migrate, and reconstruct hepatic sinusoids [20]–[23]. As reviewed recently, following CCl_4_-induced injury, endothelial cells of the liver portal vein contract with lumen constriction, and liver tissue becomes ischemic and hypoxic [24], [25]. Infusion of EPCs or VEGF can enhance neovascularization, relieving portal pressure and eventually ameliorating the fibrosis [26], [27]. In the present research, HD culture of bone marrow cells enriched EPCs more than RD culture (Fig. 1). In addition, IHC of frozen sections confirmed that more blood vessels were formed in the liver after treatment with HD cultured cells [15]. The enhancement of capillary density was coinciding with the treatment by EPCs Prevention of liver fibrosis and liver reconstitution of DMN-treated rat liver by transplanted EPCs [28]. Interestingly, these findings are conflicting with the reports that liver fibrogenesis and angiogenesis develop in parallel during progression towards cirrhosis [29], [30]. It has been reported that the drugs that specifically inhibit angiogenesis could reduce hepatic fibrosis [29], [30]. However, other studies showed that an inhibition of angiogenesis could even worsen fibrosis [31], [32]. Theoretically, angiogenesis is important for the tissue repair. Therefore, in this study, the enhanced blood supply could prevent injury, stimulate regeneration, and inhibit fibrosis.

The basic idea of HD culture is to maintain cell-cell interactions in culture, which is a well-known factor of the stem cell niche *in situ* [33]. Theoretically, besides EPCs, other stem cells, including HSCs and MSCs from the bone marrow, might also be expanded in this culture. The higher expression levels of CD34, CD29, and CD90 supported this (Fig. 1). The osteogenic and chondrogenic potential of HD cultured cells has been shown (unpublished data), while the hematogenic potential of these cells is still under investigation. HSCs and MSCs are cell types shown to possess antifibrogenic potential in liver injury. Comparing transplantation of purified cell populations, transplantation of a mix population can enhance tissue repair in many cell therapy models [34]–[36]. This is likely because of the different role of cells in one physiological and pathological process. For example, EPCs require the presence of MSCs to enhance new blood vessel formation [37]. Therefore, it is not surprising that an enhanced antifibrogenic effect was achieved by treatment with HD cultured cells containing mixed stem cell populations. In addition, growth factors favored for liver regeneration were highly expressed in this culture, which also supports better outcome of this treatment.

## Conclusions

Taken together, we demonstrated that HD cultured bone marrow cells played a more effective role in amelioration of rat liver fibrosis, functional recovery, and hepatic regeneration. This simple and cost-effective culture system provides an effective way for expansion of bone marrow cells for future clinical applications.
